# Aberrant Expression of Syndecan-1 in Cervical Cancers

**DOI:** 10.1007/s12253-020-00816-0

**Published:** 2020-05-10

**Authors:** Katalin Karászi, Renáta Vigh, Miklós Máthé, Alexandra Fullár, Lászlóné Oláh, Tibor Füle, Zoltán Papp, Ilona Kovalszky

**Affiliations:** 1 1st Department of Pathology and Experimental Cancer Research, Semmelweis University, Üllői út 26, Budapest, H-1085 Hungary; 2 1st Department of Obstetrics and Gynecology, Semmelweis University, H-1082 Budapest, Hungary; 3 Maternity Obstetrics and Gynecology Private Clinic, H-1126 Budapest, Hungary

**Keywords:** Syndecan-1, Cervical cancer, Survival analysis, Cancer-associated fibroblasts, Extracellular matrix remodeling

## Abstract

Syndecan-1, is a transmembrane heparan/chondroitin sulfate proteoglycan necessary for cell-cell and cell-matrix interactions. Its decreased level on the cell surface correlates with poor prognosis in several tumor types. Aberrant stromal localization of syndecan-1 is also considered an unfavorable prognostic factor in various human malignancies. In the presented work the question was addressed if changes in syndecan-1 expression are related to the prognosis of cervical cancer. Immunohistochemistry for syndecan-1 extracellular domain was performed on surgical specimens of primary cervical cancer. To follow the communication between tumor cells and stromal fibroblasts, their mono-and co-cultures were studied, detecting the expression of syndecan-1, smooth muscle actin, vimentin, and desmin. Immunohistochemistry of tumorous specimens revealed that while cell surface syndecan-1 expression was reduced on cancer cells, it appeared on the surface of tumor-associated fibroblasts. Until year 7, the cohort with high cell surface syndecan-1 expression had significantly longer survival. No difference in the same time-period could be detected when stromal syndecan-1 expression was analyzed. *In vitro* analysis revealed, that tumor cells can induce syndecan-1 expression on fibroblast, and fibroblasts showed that fibroblast-like cells are built by two cell types: (a) syndecan-1 positive, cytokeratin negative real fibroblasts, and (b) syndecan-1 and cytokeratin positive epithelial-mesenchymal transformed tumor cells. Syndecan-1 on the surface of cancer cells appears to be a positive prognostic marker. Although syndecan-1 positive fibroblasts promote tumor cell proliferation *in vitro*, we failed to detect their cancer promoting effect *in vivo*.

## Introduction

In spite of recent advances in prevention, early diagnosis, effective screening and treatment programs, cervical cancer is still associated with the fourth highest mortality rate in women oncology worldwide [1, 2].

Stromal cells and extracellular matrix (ECM) elements have tremendous impact on the nature of cancer. The tumor cells remodel their local surrounding environment by the production of stimulatory factors and cytokines forming the tumorous neostroma which contains cancer-associated fibroblasts (CAFs), endothelial cells and inflammatory cells [3]. CAFs producing matrix proteins in increased quantity and altered quality are important players in ECM remodeling [4]. ECM plays a key role in tumor cell invasion and metastasis. By producing metalloproteases the invading cells degrade and pass through the basement membrane. This event opens up the gate for further migration of tumor cells through the remodeled interstitial matrix [5, 6].

Syndecans, a four-member family of proteins belonging to the group of integral membrane heparan sulfate proteoglycans (HSPGs), participate in cell-cell recognition and cell-matrix interactions [7–10]. Syndecan-1 (sdc1), a typical component of the epithelial cell, acts as growth factor reservoir or co-receptor for stimulatory signal transduction on the cell surface [7, 9–11]. This protein molecule has a short cytoplasmic domain with four phosphorylation sites, a transmembrane and an extracellular domain; the latter bearing the heparan sulfate and chondroitin sulfate glycosaminoglycan chains responsible for growth factor binding [7, 12–15].

The role of syndecan-1 in various human tumors has been extensively investigated. Decrease of epithelial cell membrane syndecan-1 expression, observed in precancerous lesions of the oral cavity and uterine cervix, is regarded as a step towards malignant transformation [16–18]. Later on during the progress of tumor microevolution, the malignant clone apparently takes advantage of the loss of cell surface syndecan-1, as observed through the connection between decreased cell surface syndecan-1 levels and unfavorable prognosis in head and neck tumors and gastric cancers [19–21]. The extent of membrane syndecan-1 loss correlates with the grade of cervical cancers and also presumably with the presence of lymph node metastasis, without showing correspondence with other main clinical parameters [20, 22–24].

Normally, syndecan-1 expression is limited to the epithelia and certain B-lymphocytes, including immature B-cells and plasma cells [25]. Nevertheless, fibroblasts in the stroma of tumors may also express syndecan-1 [26]. This tumor-induced aberrant stromal syndecan-1 expression is an unfavorable prognostic factor in mammary and gastric malignancies, in head and neck squamous cell cancers as well as other tumors [27–33].

Here, we investigated the significance of pathological syndecan-1 expression and localization in cervical cancers and correlated syndecan-1 presence with the 15-year survival rate.

## Materials and Methods

### Sample Collection

Paraffin sections of Wertheim hysterectomy for survival study were received between 2000 and 2002 from the 1st Department of Obstetrics and Gynecology of Semmelweis University (Budapest, Hungary). From 2004 fresh surgical specimens used for tissue microarray and tissue culture work obtained from radical Wertheim hysterectomy were sent for routine pathology service to the 1st Department of Pathology and Experimental Cancer Research from the Maternity Obstetrics and Gynecology Private Clinic and the 1st Department of Obstetrics and Gynecology of Semmelweis University (Budapest, Hungary). Surgical samples were collected after written informed consent and used according to the instructions of Semmelweis University Regional and Institutional Committee of Science and Research Ethics (TUKEB permit number: 95/1999). Specimens and data were stored anonymously. The study conforms to the standards set by the Declaration of Helsinki. Table 1 summarizes the clinical data of the patients classified into 3 study groups: (1) survival study group; (2) tissue microarray (TMA) study group; and (3) primary culture study group.

**Table 1:** Clinical data of study groups

	Survival study		TMA study		Primary culture study	
Number of cases	55		58		17	
Median age (years)	47 (25–77)		45 (31–71)		51(36–70)	
Histology	Number	%	Number	%	Number	%
Adenosquamous carcinoma	1	1.8	5	8.6	1	5.9
Adenocarcinoma	0	0.0	7	12.1	1	5.9
Squamous cell carcinoma	54	98.2	43	74.1	15	88.2
Dysplasia	0	0.0	1	1.7	0	0.0
No tumor	0	0.0	2	3.4	0	0.0
FIGO Stage						
IA	2	3.6	1	1.7	-	-
IB	15	27.3	16	27.6	1	5.9
IIA	12	21.8	10	17.2	1	5.9
IIB	23	41.8	14	24.1	-	-
IIIB	3	5.5	-	-	-	-
No data	0	0.0	17	29.3	15	88.2
Grade						
1	19	34.5	13	22.4	-	-
2	30	54.5	23	39.7	-	-
3	6	10.9	2	3.4	-	-
No data	0	0.0	20	34.5	17	100.0
Metastasis	22	40.0	27	46.6	7	41.2
HPV status						
Only HPV16	27	49.1	17	29.3	-	-
Only HPV18	2	3.6	2	3.4	-	-
Only HPV33	0	0.0	0	0.0	-	-
HPV16 + 18	12	21.8	5	8.6	-	-
HPV16 + 33	7	12.7	1	1.7	-	-
HPV18 + 33	1	1.8	0	0.0	-	-
HPV16 + 18 + 33	5	9.1	3	5.2	-	-
HPV negative	1	1.8	8	13.8	-	-
No data	0	0.0	22	37.9	17	100.0
No survival data	4	7.3	-	-	-	-

### Tissue Samples, Tissue Microarray and Immunohistochemistry (IHC)

The surgical specimens were fixed in formalin and embedded in paraffin (FFPE). Tissue sections were stained with hematoxylin and eosin (H&E) for histopathological examination.

Fifty-five women with cervical cancer who underwent radical hysterectomy in 2000–2002 were chosen into the survival study group. All cases had previously been analyzed for HPV genotypes.

In addition, 58 cervical cases were enrolled between 2004 and 2012 into the TMA study group. Representative normal and tumorous areas were selected for the construction of TMAs, which contained 2 mm cores in diameter of each case.

Syndecan-1 (clone BB4) staining of the survival study group and its evaluation were followed as previously reported [32]. Briefly, independent observers manually scored membrane syndecan-1 based on intensity levels ranging from 0 to 2 + and absence or presence of stromal syndecan-1 in tumor areas twice. As later on BB4 clone was not available anymore, syndecan-1 expression was tested on still available sections with the clone MI15, as well.

Syndecan-1 (clone MI15) and vimentin immunostainings of TMAs were carried out using a Leica BOND-MAX™ autostainer (Leica GmbH, Nussloch, Germany), according to the manufacturer’s instructions [34]. Primary and secondary antibodies are listed in Table 2. Syndecan-1 (clone MI15) and vimentin primary antibodies are used in routine diagnostic use.

**Table 2:** Antibodies used

Antibody		Host species, isotype	Manufacturer*	Cat. No.	Dilution IHC/IF
Primary	Vimentin	Mouse monoclonal IgG, clone V9	Dako	M0725	1:300
	Cytokeratin	Mouse monoclonal IgG, clone AE1/AE3	Dako	M3515	1:100
	Smooth Muscle Actin (SMA)	Mouse monoclonal IgG, clone 1A4	Dako	M0851	1:200
	Desmin	Mouse monoclonal IgG, clone D33	Dako	M0760	1:100
	Syndecan-1 (sdc1)	Mouse monoclonal IgG, clone MI15	Dako	M7228	1:100
	Syndecan-1 (sdc1)	Mouse monoclonal IgG, clone B-B4	AbD Serotec	MCA681F	1:500
	Pan cytokeratin FITC labeled	Mouse monoclonal IgG, clone LP34	Dako	F0859	1:100
	CD138 Alexa Fluor® 647 labeled	Mouse monoclonal IgG, clone B-B4	AbD Serotec	MCA681A647	1:100
Secondary	Anti-mouse IgG/Biotin	Goat polyclonal	DakoCytomation	E0433	1:200
	Alexa Fluor® 568 anti-mouse IgG (H + L)	Donkey polyclonal	Invitrogen	43497A	1:200
	Alexa Fluor® 488 anti-mouse IgG (H + L)	Donkey polyclonal	Invitrogen	A21202	1:200
	Anti-Mouse IgG TRITC	Goat polyclonal	Sigma-Aldrich	T5393	1:100

IHC: immunohistochemistry, IF: fluorescent cytochemistry

*Dako/DakoCytomation, Agilent Technologies, Inc., Santa Clara, CA. USA; Invitrogen by Life Technologies. Carlsbad. CA. USA; Sigma-Aldrich/Merck, St. Louis, MO, USA; AbD Serotec, Kidlington, UK

Stained TMA slides were digitally scanned with a Scan Scope CS2 (Aperio Technologies Inc., Vista, ca., USA). Staining intensities were analyzed by Algorithm Positive Pixel Count Analysis software (Aperio Technologies Inc.), then percentage of positive and negative pixels were evaluated.

### DNA Extraction and Determination of HPV Status

DNA was isolated using High Pure PCR Template Preparation Kit (Roche Diagnostic GmbH, Mannheim, Germany) following the manufacturer’s instructions. DNA concentration and purity were measured with a NanoDrop 1000 spectrophotometer (Thermo Fisher Scientific, Waltham, MA, USA).

Type-specific determination of HPVs was carried out by nested PCR as described previously [35].

### Raw Materials

The raw materials and devices used in cell culture were from Sigma-Aldrich Co. (St. Louis, MO, USA) and SARSTEDT AG&Co (Nümbrecht, Germany). Reagents used in the experiments were purchased from Sigma-Aldrich Co. and Merck (Darmstadt, F. R. Germany).

### Generation of Primary Cell Cultures

Fibroblasts from normal (NF) and tumorous (CAF) regions of uterine cervix not used for diagnosis were obtained from explant cultures. These surgical specimens were excised and cut into small pieces and placed into six-well tissue culture dishes containing AmnioGrow Plus medium (CytoGen GmbH, Sinn, Germany), optimized for development of primary cell culture. After the third passage, growing fibroblasts were routinely transferred into DMEM-low glucose medium supplemented with 10% fetal bovine serum (FBS), 2 mM L-glutamine, 100 unit/mL penicillin and 100 µg/mL streptomycin. Purity of the fibroblast cultures was tested by vimentin and cytokeratin fluorescent immunostaining. We established 22 primary cervical cultures (7 NFs + 15 CAFs) from 17 patients for immunocytochemistry examinations (Table 1, primary culture study group). Primary cultures were used for research until 10th passage. Fibroblasts were vimentin positive and pan-cytokeratin negative cells displaying spindle-like morphology, with elongated, oval nuclei.

HPV16-positive CSCC1 (cervical squamous cell carcinoma 1) cervical cancer cell line, derived from a case of squamous cervical cancer, was a kind gift from A. Gorter (Leiden University, Leiden, the Netherlands) [36]. These cells exhibit a clear epithelial morphology and form nests when grown in monoculture. They express pan-cytokeratin but lack vimentin. CSCC1 cells were routinely cultured in RPMI-1640 medium supplemented with 10% FBS, 2 mM L-glutamine, 1 mM sodium pyruvate, 100 unit/mL penicillin and 100 µg/mL streptomycin. All cell lines and primary cells were cultured in a humidified 95% air / 5% CO_2_ incubator at 37 °C.

Fibroblasts were cultured either as monocultures or direct co-cultures with CSCC1.

### Direct Co-culture System

Direct co-culturing allowed physical interaction between fibroblasts and tumor cells. Accordingly, NF and CSCC1 cells were seeded alone (5 × 10^5^ cells/culture dish density) or in the same culture dish together (NF + CSCC1; 5 × 10^5^-5 × 10^5^ cells/culture dish density) in a 1:1 (v/v) mixture of DMEM-low glucose and RPMI-1640 supplemented with 10% FBS, 2 mM L-glutamine, 100 unit/mL penicillin and 100 µg/mL streptomycin.

### Immunocytochemistry

Forty-eight hours after seeding cells from monoculture and direct co-culture model systems grown on coverslips were captured using Olympus CK2 inverted phase microscope equipped with an Olympus DP50 CCD camera (Olympus Corporation, Tokyo, Japan). Then immunofluorescence staining was performed on methanol-acetone-fixed glass coverslips according to standard protocols [37]. In case of multiple labeled fluorescent staining, vimentin or desmin was labelled with TRITC, then pan cytokeratin conjugated to FITC and Alexa Fluor® 647 labeled syndecan-1 were used. Primary and secondary antibodies are detailed in Table 2. Nuclei were stained with 1 µg/mL DAPI in PBS for 5 min except by triple-color staining. Photographs were taken with a Nikon Eclipse E600 microscope (Nikon Corporation, Tokyo, Japan) operated by the Lucia Cytogenetics version 1.5.6 program (Laboratory Imaging, Praha, Czech Republic) or MRC-1024 confocal laser scanning microscope (Bio-Rad Laboratories GmbH, Munich, Germany).

### Statistical Analysis

Data were analyzed using Microsoft Excel v.2016 (Microsoft Corp., Redmond, WA, USA) and GraphPad Prism 8 (GraphPad Software, La Jolla, ca., USA). The expression differences between normal cervix and cervix carcinoma in case of TMAs were analyzed by nonparametric Mann–Whitney test. Correlation analysis between IHC scores and clinicopathologic data of patients were performed with a nonparametric Spearman rank-order correlation test. Survival curves were analyzed using the Kaplan-Meier method and differences between survival curves of different groups were defined by the log-rank test. A *p* value of 0.05 was set as the threshold for statistical significance.

## Results

### Expression of Cell Surface Syndecan-1 is Related to Survival

A decrease in cell surface syndecan-1 (sdc1) expression was detectable in the majority of the tumors examined. No membrane reaction comparable in intensity with normal epithelial cells was detected. A moderately decreased reactivity (2+) was recorded in 13/55, a strongly decreased (1+) in 20/55 and no reaction (0) in 22/55 cases. The metastases present in the lymph nodes also showed decreased syndecan-1 membrane positivity as well.

**Fig. 1:**
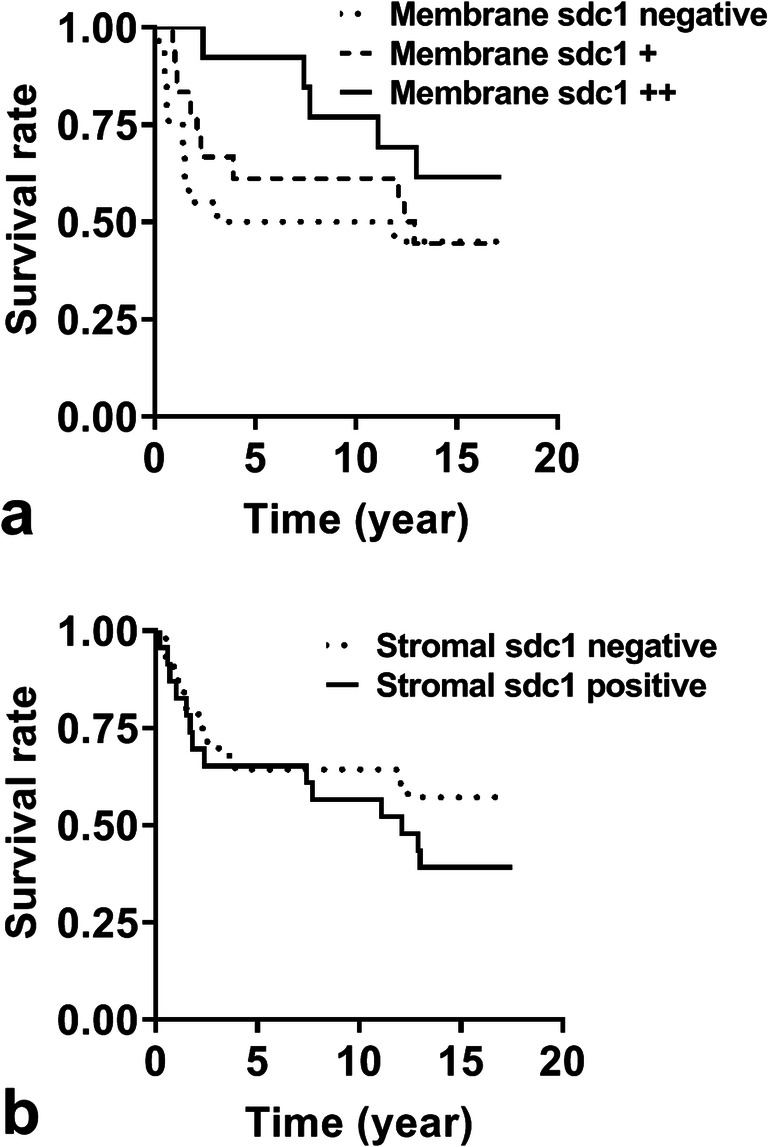
Cell surface syndecan-1 expression provides survival benefit for seven years . Kaplan–Meier 15-year survival curves of patients with cervical cancer grouped by the presence or absence of membrane (**a**) and stromal (**b**) syndecan-1 (survival study group). Sdc1: syndecan-1

Similar to earlier studies, the degree of reduction of membrane syndecan-1 expression followed the histological grade, i.e. more differentiated tumors expressed more syndecan-1 (Spearman correlation: *p* = 0.0055, r=-0.3696). Loss of membranous syndecan-1 expression associated with higher mortality rate when the postoperative survival time was elongated (51/55) (Fig. 1a). The 5, 10 and 15-year overall survivals were 64.7% (log rank test: *p* = 0.0374), 60.8% (log rank test: *p* = 0.1721) and 49% (log rank test: *p* = 0.3663). The promotion of survival was significant until the seventh year.

### Effect of Stromal Syndecan-1 Expression On Survival

In 26/55 (47%) cervical tumors, abnormal syndecan-1 localization was found in the stroma, most probably due to aberrant expression of this proteoglycan by stromal fibroblasts. Moreover, the lymph node metastases of these tumors preserved their inductive potential on stromal cells, as fibroblasts detected in metastases were also positive for syndecan-1. It was observed that the more of the syndecan-1 was localized in the stroma, the less of it was found on the cancer cells. However, although it seems likely that aberrant expression of syndecan-1 favors tumor progression, analysis of 51/55 patients in the survival study group revealed lack of significance between stromal syndecan-1 expression and survival (log rank test: *p* = 0.2765; Fig. 1b).

### Variations of Syndecan-1 and Vimentin Expression in Cervical Cancer

**Fig. 2:**
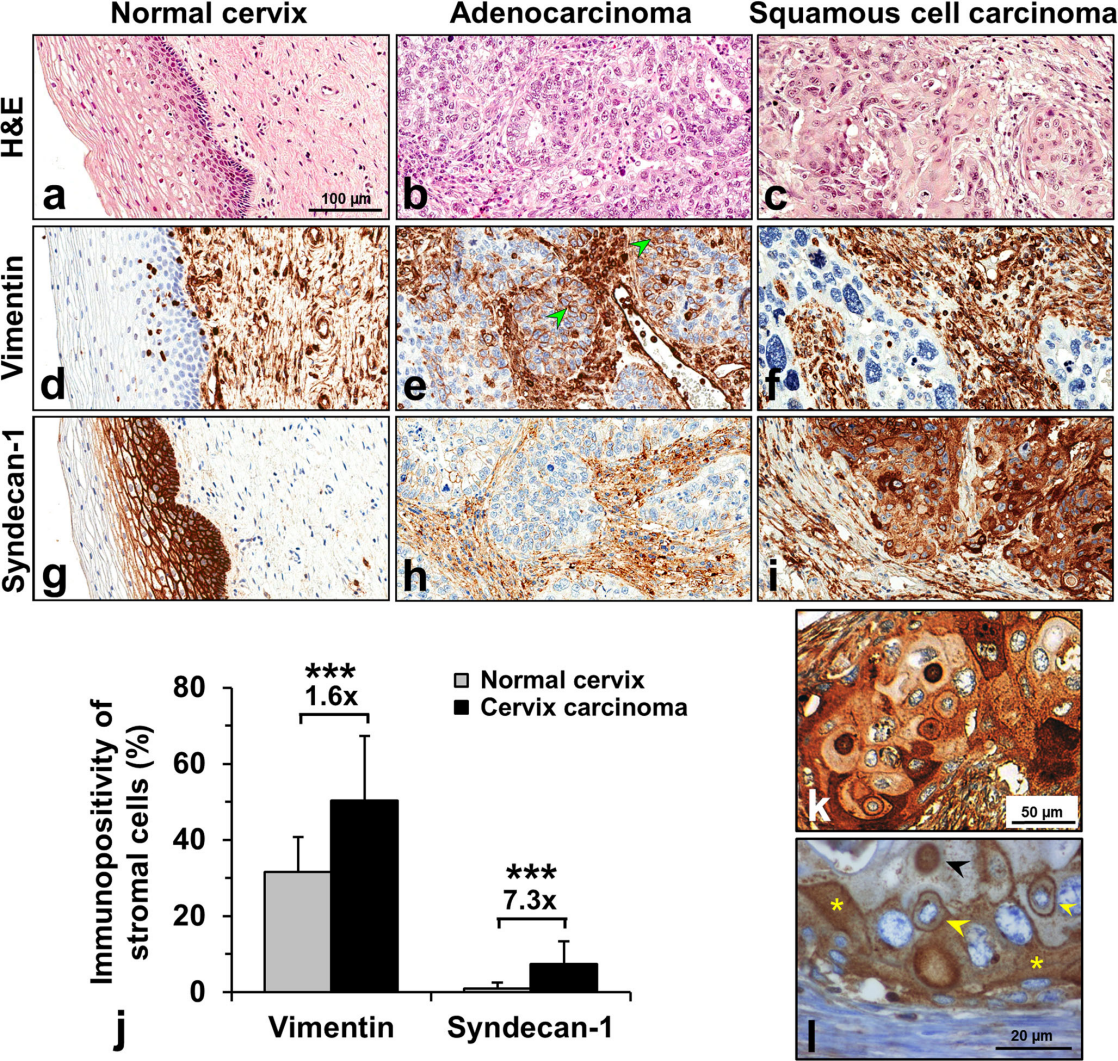
Expression of vimentin and syndecan-1 in normal cervix and cervical cancer. H&E staining of normal cervix, adenocarcinoma and squamous cells of the cervix (**a-c**) (TMA study group). Vimentin was present as homogenous staining in the connective tissue of normal cervix (**d**), the reaction is distributed in the tumorous stroma in a similar manner (**e,f**). In addition, cytoplasmic vimentin positivity can be detected both in adenocarcinoma (green arrows) (**e**) and squamous cell carcinoma (data not shown). Strong membranous syndecan-1 reaction is detectable in normal cervical epithelium (**g**), whereas aberrant stromal syndecan-1 positivity appears in cervical cancer (**h,i**). Densitometric evaluation showed that both vimentin and syndecan-1 immunostainings were significantly increased in the stromal compartment of cervical cancer tissues (black column) compared to normal tissues (gray column). The signal positivity of stromal cells is shown as a proportion of the total measured area (**j**). Specificity of immunoreaction of both syndecan-1 antibodies on the same squamous cell carcinoma sample of survival study group presented on **k** (clone MI15) and **l** (clone BB4). Cell surface syndecan-1 seems to translocate to the nucleus (black arrow), the perinuclear membrane (yellow arrow) and the cytoplasm (yellow star) abnormally (**k**). Representative images of same TMA cores (**a-i**) or sample (**k,l**), hematoxylin counterstain, 100x magnifications with scale bar 100 µm (**a-i**); 200x magnifications with scale bar 50 µm (**k**); 400 x magnification with scale bar 20 µm (**l**), stars indicate significance: ***p < 0.001

Immunostaining of TMA allowed the simultaneous examination of peritumoral, seemingly normal and tumorous cervix in 58 cases. In addition to syndecan-1, the expression of vimentin was also detected. Stromal cells of tumorous area contained more vimentin than those from non-tumorous regions (1.6-fold, Mann-Whitney test: *p* < 0.0001) (Fig. 2d-f,j). Moreover, indicating epithelial-mesenchymal transition (EMT), vimentin occurred in the cytoplasm of several tumor cells (Fig. 2e). Syndecan-1 expression of normal squamous epithelial cells showed similar trends to those of the survival study group (Fig. 2g). Similarly to the survival study, syndecan-1 intensity of tumorous membrane decreased and its presence in the interstitial stroma could be detected (Fig. 2h,i). The intensity of stromal syndecan-1 immunostaining was 7.3-fold higher (Mann Whitney test: *p* < 0.0001) in cervical cancer than in normal tissues (Fig. 2j). Few samples from survival study group were stained by both syndecan-1 antibodies showing the same localization on the same samples (Fig. 2k,l). Furthermore, Fig. 2i,k and l are good examples for altered localization of syndecan-1 in cancer cells showing the proteoglycan in the cytoplasm, infrequently in the nucleus and the perinuclear membrane of cancer cells.

### Characteristics of Normal and Tumorous Fibroblasts in Tissue Cultures

Seven NF and 15 CAF cervical primary cultures have been established, containing 3 NF–CAF matched primary culture pairs derived from the same 3 patients.

**Fig. 3:**
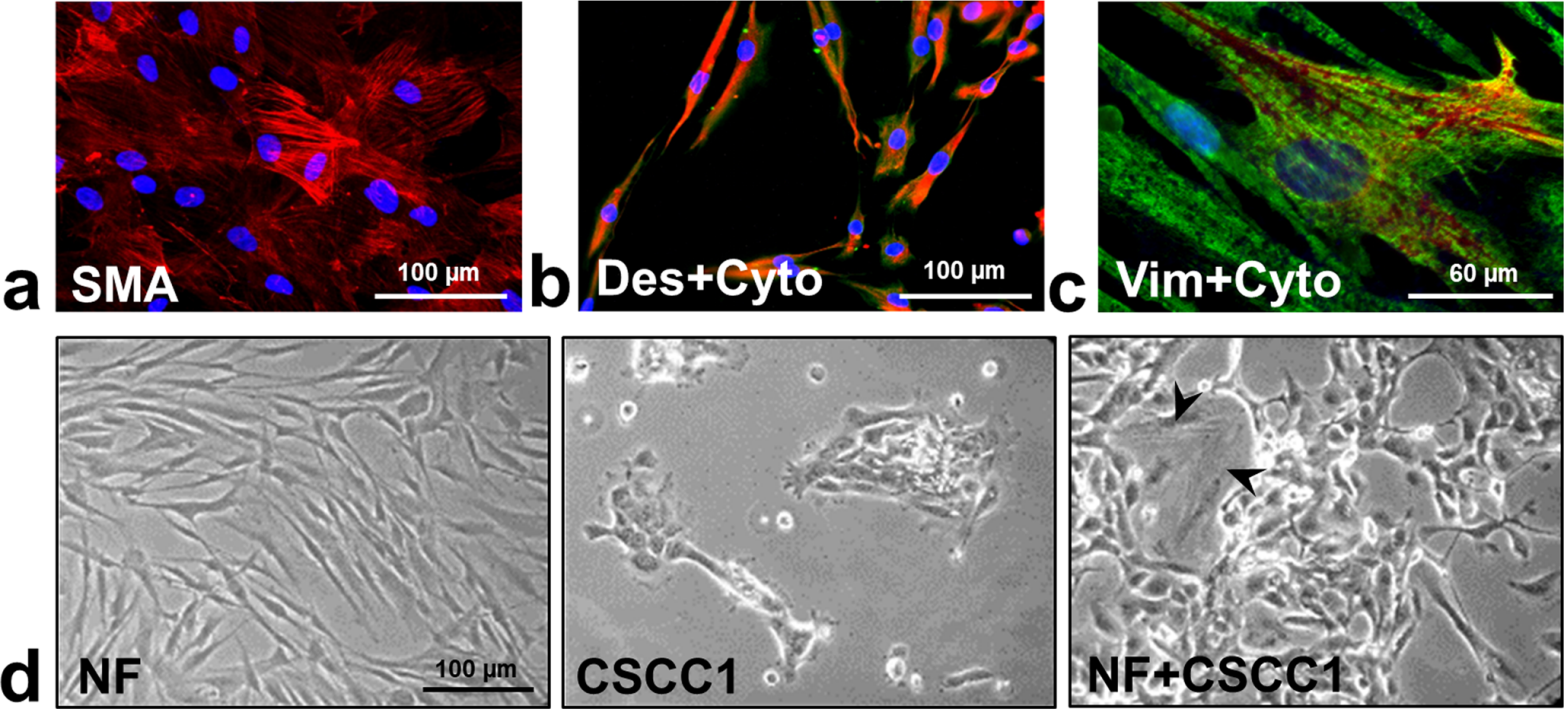
Fibroblasts and their effect on the proliferation of CSCC1 cells. Most of the fibroblast primary cultures expressed SMA (**a**). Some cells showing fibroblast morphology with desmin positivity (red) also expressed epithelial marker cytokeratin (green) (**b**) or with vimentin positivity (green) and cytokeratin positivity (red) (**c**). Nuclei were stained with DAPI. CSCC1 cells grew much faster in the presence of NFs (black arrow) than alone 48 h after seeding; equal number of cells were seeded (**d**); 200x magnifications with scale bar 100 µm, 600x magnifications with scale bar 60 µm

All of 22 fibroblast primary cultures showed the presence of vimentin and desmin. From those, 17 expressed SMA in various amounts indicating that these cells are activated myofibroblasts (Fig. 3a). Syndecan-1 was detected in 14 primary cultures most of them being CAFs (13/15). In case of NF-CAF matched pairs, all NFs were negative, and all CAFs expressed syndecan-1.

In addition to cytoplasmic desmin/vimentin, few cells from 6 CAF primary cultures also expressed cytokeratin (6/15) presenting EMT of tumor cells (Fig. 3b,c). Three of them also expressed syndecan-1 in less than 3% of cytokeratin positive cells as we published earlier [38]. In co-culture the presence of NFs stimulated the proliferation of CSCC1 cells compared to their monoculture (Fig. 3d).

**Fig. 4:**
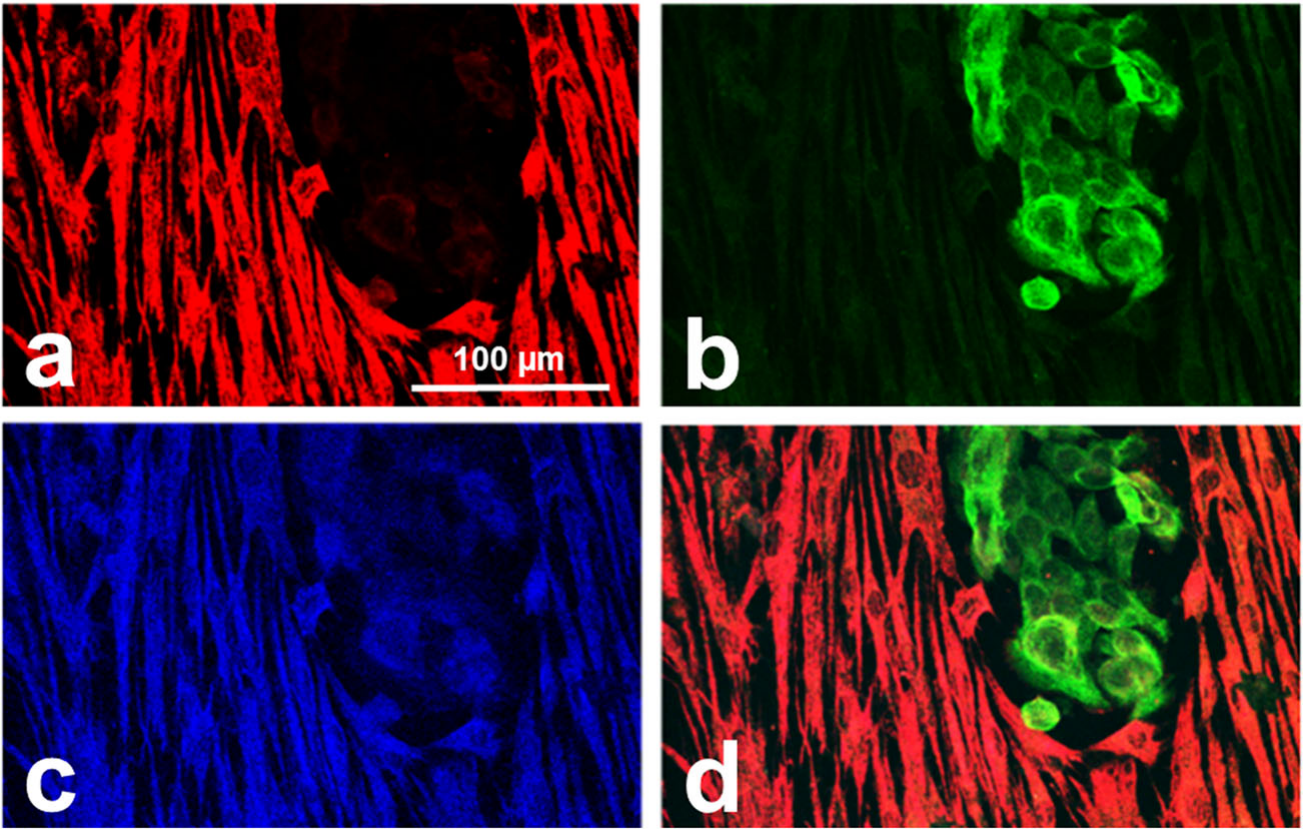
Tumor-induced aberrant syndecan-1 positivity of cervical fibroblasts. In monoculture the presented NFs were syndecan-1 negative, however in direct co-culture with syndecan-1-positive CSCC1, NFs also expressed syndecan-1 (blue, **c**) in addition to fibroblast marker vimentin (red, **a**). CSCC1 showed presence of the epithelial marker cytokeratin (green, **b**) and the lack thereof for vimentin. Merged image of triple-color immunofluorescence (**d**); 200x magnifications, scale bar 100 µm

Co-cultivation of NFs with CSCC1 cells induced syndecan-1 expression in previously syndecan-1-negative fibroblasts (Fig. 4).

## Discussion

Previous studies on the role of syndecan-1 in tumors mainly focused on the changes of this HSPG taking place on the surface of tumor cells. In support of earlier reports, we observed serious abnormalities in the expression of syndecan-1 in invasive cervical cancers. In comparison with the normal epithelium, tumor cell membrane syndecan-1 immunoreaction was moderately or strongly decreased in the majority of the cases; moreover, its occasional disappearance was also observed. Higher syndecan-1 expression was accompanied with significantly better survival until the 7th year indicating that it can be considered as a prognostic marker. Statistical significance was lost in the following 8 years, presumably due to decreased number of cases. This is in an agreement with long-term survival study of Numa et al. [24]. We attempted to utilize the TMA group for survival analysis to increase the number of cases of survival study, however it seems that the small areas present in the TMA are not representative enough to gain reliable results. Henceforward, using the TMA platform and the computed analysis we compared the stromal syndecan-1 and vimentin expression on normal and tumorous areas.

In recent years, increasing interest has been directed towards the ectopic stromal expression of syndecan-1. Increasing evidence indicates that human tumor cells are capable of inducing stromal syndecan-1 expression which represents a distinct subgroup with worse life expectancy [21, 28]. In our study syndecan-1 immunoreaction was noticeable in vimentin-positive mesenchymal cells, identifying these cells as the source of stromal syndecan-1. Aberrant, tumor-induced stromal expression of syndecan-1 both in squamous cell and adenocarcinomas, as recorded in our specimens, is a novel finding in cervical cancer. Also, to confirm the specificity of this abnormal expression we used two syndecan-1 antibodies receiving the same abnormal expression. The stromal syndecan-1 expression was regularly seen in combination with the reduced membranous syndecan1 positivity of the tumor cells. In spite of that, we failed to confirm significant relationship between stromal syndecan-1 expression and patient survival.

A rather general observation is that in the complex relationship between the stroma and the tumor cells syndecan-1 acts as a mediator of reciprocal interactions [33]. The heparan sulfate glycosaminoglycan chains of this proteoglycan intensively influence the quantity and localization of growth factors and cytokines in the tumor microenvironment. An excess of growth factors, bound by heparan sulfate chains of syndecan-1, abnormally expressed on the surface of stromal cells may lead to aggressive clinical tumor phenotype. This hypothesis has been supported by *in vitro* studies, in which ductal mammary tumor cells and fibroblasts were co-cultured [33]. Further support comes from a relevant *in vivo* study [39]. Our previous work demonstrated that remodeling of ECM by fibroblasts promotes cervical cancer progression [38]. Now, our culture models confirmed that syndecan-1 expression by stromal cells is a typical feature of fibroblasts isolated from cervical cancers. Most of the CAFs presented syndecan-1 positivity in monoculture. Furthermore, their direct contact with cancer cells induced the production of syndecan-1 in normal fibroblasts that lacked syndecan-1 expression in monoculture. Syndecan-1-expressing fibroblasts reorganize the ECM promoting adhesion and directional movements of tumor cells during migration [40]. As even NFs started to express syndecan-1 in direct co-culture indicates that tumor cells are responsible for the induction of this proteoglycan. Higher syndecan-1 levels, in turn, accelerate the proliferation of the tumor cells, thus constituting a positive feedback loop [41, 42].

The question has to be addressed if syndecan-1 expression promotes the aggressive phenotype of EMT transformed tumor cells present in the stroma. We determined the proportion of vimentin-syndecan-1-cytokeratin positive cells in our primary fibroblast cultures. Among those six contained cytokeratin positive cells but they were syndecan-1 negative in 3 cultures. The proportion of cells with cytokeratin/syndecan-1 positivity was below 3% in the remaining three cases. This result indicates, that syndecan-1 does not enhance the aggressive phenotype of EMT transformed tumor cells of cervix, which can be accomplished by the classical markers of EMT.

In addition to epithelial-mesenchymal switch, other abnormalities also can be found in cervical cancers. Impaired secretion of the proteoglycan results in cytoplasmic localization, and syndecan-1 presence can be detected in the cell nuclei as well. These findings are supported by multiple studies investigating other types of cancer [43–46]. Kim et al. scoring the cytoplasmic syndecan-1 in cervical cancer found that strong syndecan-1 expression is associated with better survival [47]. Changing syndecan-1 localization from surface to cytoplasm in cancers leads to lose its original function, connecting the cell to ECM. This allows increased mobility, thus, gaining invasive and metastatic properties and increasing the number of fibroblast-like cells in the tumor microenvironment. Certainly, in our tissue culture model, double vimentin-cytokeratin immunostaining demonstrated this mesenchymal transition of tumor cells.

## Conclusions

Independent of the ultimate clinical outcome, our immunohistochemical and immunocytochemical observations confirmed that tumor-induced stromal syndecan-1 expression is a common event in human carcinomas, including cervical cancers. Although, *in vitro* studies demonstrated that proliferation of cancer cells is stimulated by stromal syndecan-1, we failed to prove its negative effects *in vivo*, most likely due to the involvement of additional regulatory factors.

## Data Availability

All data generated or analyzed during this study are included in this published article.
